# Supplementary material for: Simultaneous Electrochemical Detection of Dopamine and Tryptophan Using 3D Goethite–Spongin Composites
Source: Biomimetics (Basel). 2024 Jun 14;9(6):357. doi: 10.3390/biomimetics9060357 (PMC11202307; doi:10.3390/biomimetics9060357)
Supplement: Supplementary file 1 [file biomimetics-09-00357-s001.zip › biomimetics-2997256-SI.pdf]

biomimetics

Supplementary Data

Simultaneous Electrochemical Detection of Dopamine and Tryptophan using 3D Goethite-Spongin Composite

Sedigheh Falahi [1], Anita Kubiak [2,3], Alona Voronkina [4], Hermann Ehrlich [2,5], Yvonne Joseph [1,6] and Parvaneh Rahimi [1,6*]

1 Institute of Nanoscale and Biobased Materials, Faculty of Materials Science and Material Technology, Technische Universität Bergakademie Freiberg, 09599 Freiberg, Germany; sedigheh.falahi@doktorand.tu-freiberg.de (S.F.); yvonne.joseph@esm.tu-freiberg.de (Y.J.)
2 Faculty of Chemistry, Adam Mickiewicz University, Uniwersytetu Poznanskiego 8, 61-614 Poznan, Poland; anikub@amu.edu.pl (A.K.)
3 Center of Advanced Technology, Adam Mickiewicz University, Uniwersytetu Poznanskiego 10, 61-614 Poznan, Poland;
4 Department of Pharmacy, National Pirogov Memorial Medical University, Vinnytsya, Pyrogov Street 56, 21018 Vinnytsia, Ukraine; voronkina@vnmu.edu.ua (A.V.)
5 Faculty of Chemical Technology, Institute of Chemical Technology and Engineering, Poznan University of Technology, Berdychowo 4, 60-965 Poznan, Poland; isididae@gmail.com (H.E.)
6 Freiberg Water Research Center, Technische Universität Bergakademie Freiberg, 09599 Freiberg, Germany; Parvaneh.Rahimi@esm.tu-freiberg.de (P.R.)
* Correspondence: Parvaneh.Rahimi@esm.tu-freiberg.de; Tel.: +49-3731-39-2644

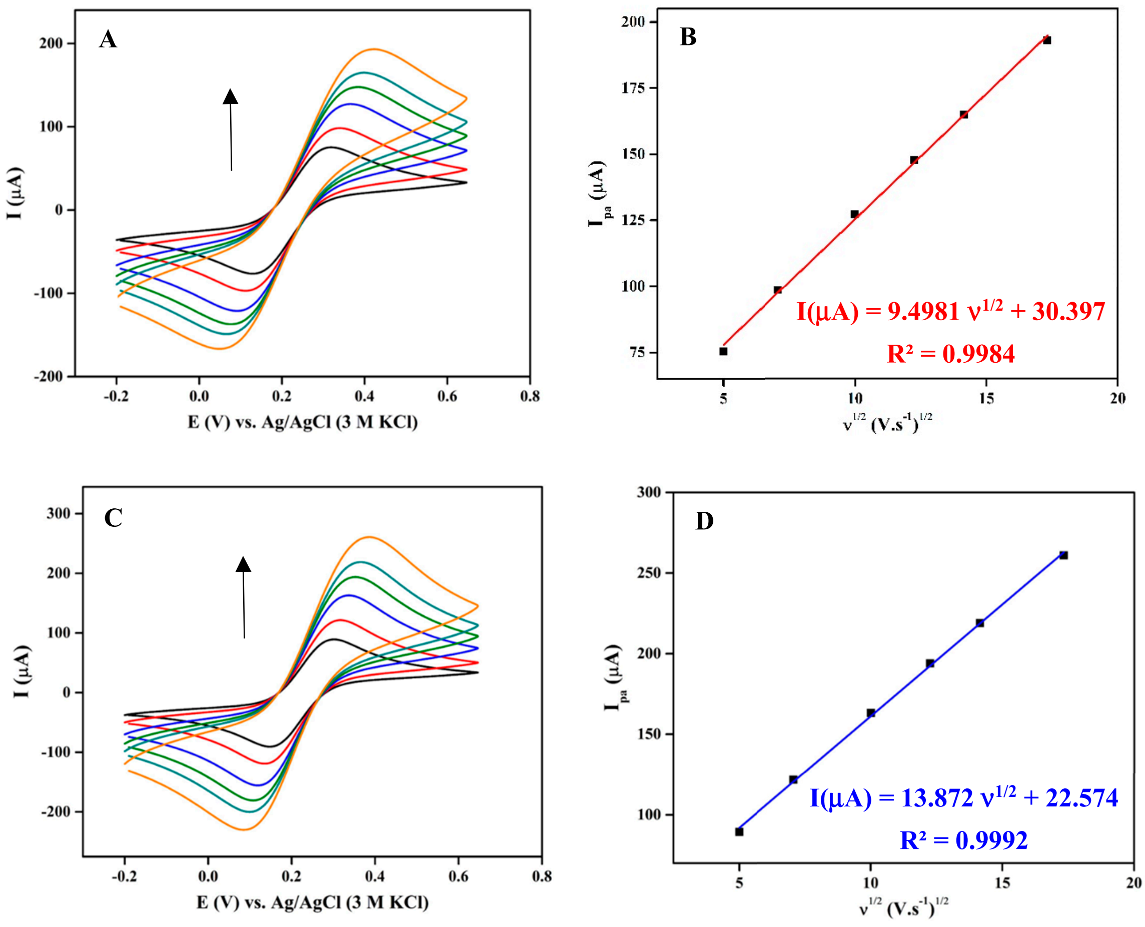

Figure S1. CVs of (**A**) CPE and (**C**) 3DGS/CPE in a solution of 0.1 M KCl containing 5 mM $[Fe(CN)_6]^{3-/4-}$ at different scan rates (25, 50, 100, 150, 200, and 300 mV.s^{-1}), and plots of I_{pa} versus the square root of the scan rate recorded at (**B**) CPE, and (**D**) 3DGS/CPE

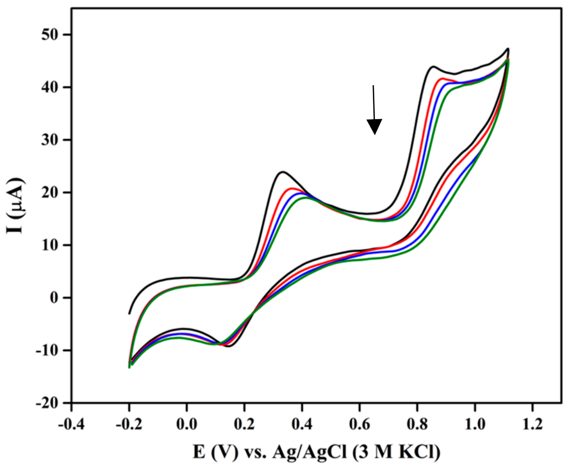

Figure S2. CVs of 3DGS/CPE in 0.1 M phosphate buffer pH 6 in the presence of 300 µM DA and TRP at a scan rate of 0.1 V.s^{-1} (5 cycles)

Table S1. Comparative study of the 3DGS/CPE performance for simultaneous detection of DA and TRP with literature reports.

Electrode	Linear range (μM)		LOD (μM)		Ref.
	DA	TRP	DA	TRP	
ZrO_2–CuO–CeO_2/CPE	0.008-185	0.009-194	0.003	0.005	24
NiO/CNT/PEDOT/GCE	0.03-20	1-41	0.026	0.21	26
CDs[1]/GCE	0.5-50	1-65	0.007	0.11	27
MIP/GCE	0.05-500	0.1-250	0.016	0.03	28
AuPdCu-rGO-MWCNTs/CPE	0.01-8.3	0.01-9.4	0.003	0.004	31
TiO_2-GR/4-ABSA[2]/GCE	1-400	1-400	0.1	0.3	32
2, 3, 4, 6, 7, 8, 9, 10-octahydropyrimido[1, 2-a] azepine/PGE[3]	1-20	1.5-750	0.05	0.05	69
NiO–CuO/GR/GCE	0.5-20	0.3-40	0.17	0.1	70
AgNPs/P(Arg)[4]-GO/GCE	0.05-50	1-150	0.01	0.122	71
β-CD[5]/CQDs[6]/GCE	4–220	5–270	0.14	0.16	72
CNOs[7]/GCE	0.01-38	1-108	0.0039	0.0087	73
Au-PDNs[8]/SPE	1-350	1-280	0.0001	0.0001	74
Ni-ZIF[9]-8/N S-CNTs/CS[10]/GCE	8-500	5-850	0.93	0.69	75
MWNTs/MGF[11]/GCE	0.3-10	5-500	0.06	0.87	76
WB-S[12] La^{3+}/TiO_2-NS[13]/SPE	0.001-900	0.001-900	0.0005	0.0008	77
PTh[14]/GPE[15]	10-180	6-180	1	0.61	78
3DGS/CPE	4-246	2-150	1.9	0.3	This work

[1]Carbon dots, [2]Aminobenzenesulfonic acid, [3]Pencil graphite electrode, [4]Poly(l-arginine), [5]Poly(β-cyclodextrin), [6]Carbon quantum dots, [7]Carbon nano-onions, [8]Polydopamine nanospheres, [9]Zeolitic imidazolate framework, [10]Chitosan, [11]Mesocellular graphene foam, [12]Woolen ball-shaped, [13]Nanostructure, [14]Polythiophene, [15]Graphite paste electrode

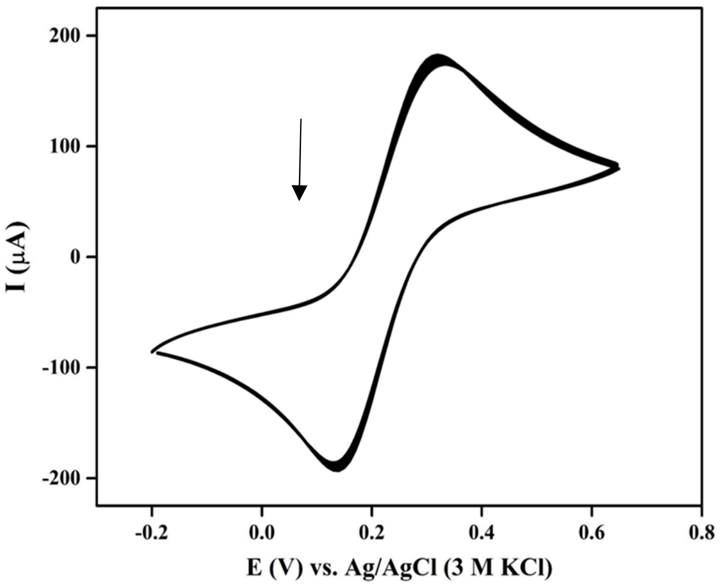

Figure S3. CVs of 3DGS/CPE in a solution of 0.1 M KCl containing 1 mM $[Fe(CN)_6]^{3-/4-}$ (100 cycles)

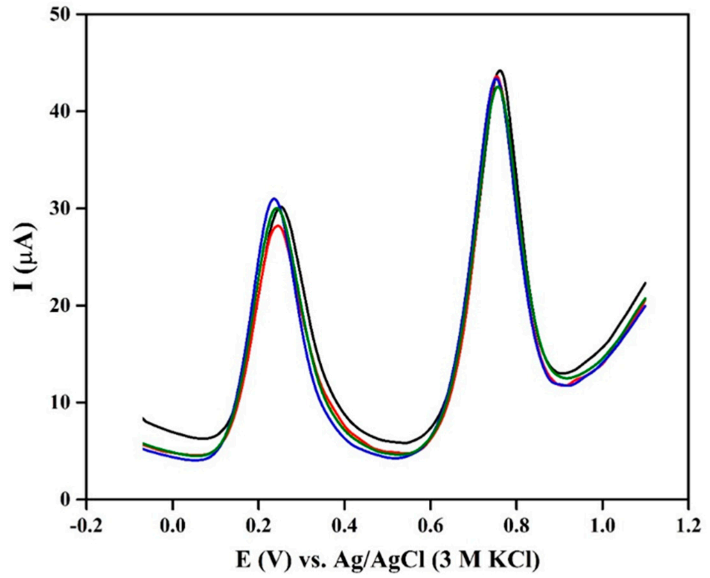

Figure S4. DPVs of 3DGS/CPE in 0.1 M phosphate buffer pH 6 containing 150 µM DA and TRP at a scan rate of 0.1 $V.s^{-1}$ (4 fresh electrodes)

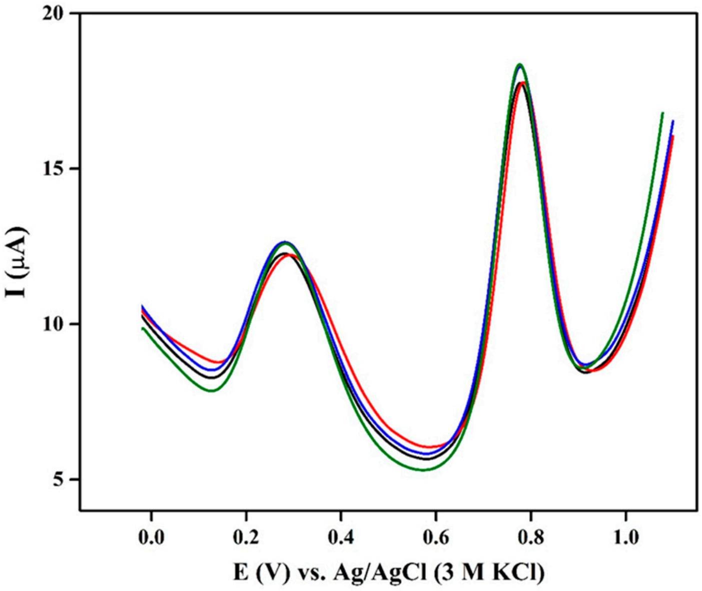

Figure S5. DPVs of 3DGS/CPE in 0.1 M phosphate buffer pH 6 containing 30 µM DA and TRP at a scan rate of 0.1 $V.s^{-1}$ (4 replicates)
